# Variation in Leaf Volatile Emissions in Potato (*Solanum tuberosum*) Cultivars with Different Late Blight Resistance

**DOI:** 10.3390/plants12112100

**Published:** 2023-05-25

**Authors:** C. A. Agho, E. Runno-Paurson, T. Tähtjärv, E. Kaurilind, Ü. Niinemets

**Affiliations:** 1 Institute of Agricultural and Environmental Sciences, Estonian University of Life Sciences, Kreutzwaldi 1, 51006 Tartu, Estonia; 2 The Centre of Estonian Rural Research and Knowledge, J. Aamisepa 1, 48309 Jõgeva, Estonia; 3 Estonian Academy of Sciences, Kohtu 6, 10130 Tallinn, Estonia

**Keywords:** volatile organic compound emission, chemodiversity, *Solanum tuberosum*, late blight resistance

## Abstract

Volatile organic compounds (VOCs) play key roles in plant abiotic and biotic stress resistance, but even for widespread crops, there is limited information on variations in the magnitude and composition of constitutive VOC emissions among cultivars with varying stress resistance. The foliage VOC emissions from nine local and commercial potato cultivars (Alouette, Sarme, Kuras, Ando, Anti, Jõgeva Kollane, Teele, 1681-11, and Reet) with medium to late maturities and varying *Phytophthora infestans* (the causative agent of late blight disease) resistance backgrounds were analyzed to gain an insight into the genetic diversity of constitutive VOC emissions and to test the hypothesis that cultivars more resistant to *Phytophthora infestans* have greater VOC emissions and different VOC fingerprints. Forty-six VOCs were identified in the emission blends of potato leaves. The majority of the VOCs were sesquiterpenes (50% of the total number of compounds and 0.5–36.9% of the total emissions) and monoterpenes (30.4% of the total number of compounds and 57.8–92.5% of the total VOC emissions). Qualitative differences in leaf volatiles, mainly in sesquiterpenes, were related to the potato genotype background. Among the volatile groups, the monoterpenes α-pinene, β-pinene, Δ^3^-carene, limonene, and *p*-cymene, the sesquiterpenes (*E*)-β-caryophyllene and α-copaene, and green leaf volatile hexanal were the major volatiles in all cultivars. A higher share of VOCs known to have antimicrobial activities was observed. Interestingly, the cultivars were grouped into high and low resistance categories based on the VOC profiles, and the total terpenoid and total constitutive VOC emission scale positively with resistance. To support and expedite advances in breeding for resistance to diseases such as late blight disease, the plant research community must develop a fast and precise approach to measure disease resistance. We conclude that the blend of emitted volatiles is a fast, non-invasive, and promising indicator to identify cultivars resistant to potato late blight disease.

## 1. Introduction

Potato (*Solanum tuberosum* L.) is the world’s third most important food crop, after rice and wheat, and it is produced on all continents except Antarctica [1]. Potato production has increased dramatically, especially in developing countries, with a global 21% increase in the past two decades, indicating its growing importance as a staple food source. For more than 160 years, potato late blight, caused by the oomycete pathogen *Phytophthora infestans* (*P. infestans*), has remained the most devastating potato disease worldwide [2,3] and other plants in the family *Solanaceae* [2,4]. Under favorable conditions, *P. infestans* can easily spread from plant to plant and can destroy entire fields [4]. The damage cost due to yield loss and disease control of the pathogen has been estimated to be over 1 billion Euros per year in Europe [5].

The application of fungicides has been implemented as one of the control strategies for late blight in potatoes. However, the constant application of fungicides has created selective pressure on the pathogen, resulting in the emergence of fungicide-resistant isolates which are becoming an important part of the pathogen populations in many countries [6]. Moreover, fungicide use has led to a high level of toxic residues in plants, which threaten both the environment and human health [7]. Thus, an environmentally sound alternative to control the pathogen is needed. Among other measures, the use of late blight-resistant potato cultivars is still considered an important aspect in the control of this disease [8]. The method requires no actions from potato growers and its use poses no harm to the environment [9]. Moreover, this approach is usually compatible with other disease management techniques [9,10]. The use of resistant varieties alone could be sufficient in managing the disease or help in reducing disease development to a tolerant level [11]. Thus, the evaluation of potato cultivars for late blight resistance is considered important in breeding for late blight resistance. Based on the intensity of symptoms, potato genotypes can differ in their levels of resistance to late blight and vary from susceptible and moderate to highly resistant genotypes [12,13]. Evaluation of foliar late blight resistance among potato genotypes has mainly been estimated using the conventional method area under the disease progress curve (AUDPC) and the relative area under the disease progress curve (RAUDPC) [14,15]. However, there are concerns about the precision of these methods [16]. Moreover, the methods are also time-consuming as a series of evaluations over time is necessary to obtain the final area value for the pathosystem [17]. The methodology also has several assumptions that make its application to the potato pathosystem difficult [18,19]. As a result, there is a renewed interest in more consistent, less time-consuming, and accurate methods for phenotyping late blight disease resistance in potatoes.

Plants exchange a huge number of volatile metabolites with their environment both aboveground and belowground [20,21]. Among plants and between varieties, the analysis of volatiles has revealed qualitative as well as quantitative variation [22]. The majority of volatile organic compounds (VOCs) known to be produced and released by plants are lipoxygenase (LOX) pathway products, phenolic compounds (methyl salicylate and benzenoids), indole, and mono-, homo-, and sesquiterpenes [23,24]. The LOX compounds are produced via the lipoxygenase pathway from free fatty acids released from plant membranes. Under different abiotic and biotic stresses, plants produce arrays of VOC that are involved both in direct and indirect defenses [23,25]. However, the VOC blend depends on the type and intensity of stress [23], and the inherent capacity of the plant to produce these [26]. By changing the volatile components and their blend ratios, plants use VOCs to stimulate plant defenses for induced and associational resistance to pathogens [27]. Several investigations have shown that some VOCs possess antimicrobial activity that inhibits pathogens’ growth and mobility within tissues and can thus act as a direct defense response [24,27,28,29,30,31,32]. It has been stated that VOC emissions not only reflect environmental suitability but also the genotypic component of the plant [33,34,35,36,37,38].

Many studies have exploited the inherent variability of constitutive VOC emissions among genotypes under natural conditions [20,39], indicating differences in specific VOC abundance between resistant and susceptible genotypes [24]. Volatile emissions are also qualitatively and quantitatively related to the level of plant resistance to biotic stress, as differences in VOC emissions exist between susceptible and resistant plant genotypes [24,40]. However, the links between genetic variations in potatoes for late blight disease resistance and constitutive volatile emission profiles remain elusive. Moreover, it is not known how VOC classes influence the genetic variation and response patterns among potato genotypes with different resistance levels to late blight disease in the field. We hypothesize that cultivars more resistant to *P. infestans* have greater VOC emissions and different VOC fingerprints. In other words, there is a response pattern between the constitutive VOC profile and late blight disease resistance. Answers to these questions would provide a platform to develop phenotypic markers for selecting late-blight-resistant potato varieties. In recent years, several pilot studies on potatoes have proven the potential usefulness of VOC monitoring but have also revealed limitations and obstacles to overcome [41]. One important consideration for a successful application is a thorough understanding of the inherent variability of VOC release under natural conditions [20]. However, establishing an accurate reference baseline of VOC emissions is not straightforward, as the quantity and composition of VOCs may vary among cultivars [20]. To gain insight into the potential of using VOC emissions to identify potato cultivars resistant to late blight and provide a benchmark for further studies, we investigated the pattern of variability of constitutive VOC emissions among potato cultivars with variations in late blight resistance.

## 2. Materials and Methods

### 2.1. Study Location and Plant Material

The experiment was carried out in the field in 2018 at the Centre of Estonian Rural Research and Knowledge (METK) in Jõgeva, Estonia, located in north-eastern Europe (58°45′ N, 26°24′ E) on clay loam (40–50% of clay classified as Calcaric Cambic Phaeozem (Loamic) soil [42]. The preceding crop in the trial area was red clover (*Trifolium pratense* L.). Seed potato tubers were planted on 21 May. The research evaluated nine (9) cultivars with different levels of resistance to late blight (Ando, Anti, Jõgeva kollane, Reet, Sarme, and Teele from METK and Alouette and Kuras from the Dutch breeding company Agrico) and one promising breeding line 1681-11 from METK. Thus, one of the main targets for the cultivar selection process was the higher field resistance to potato late blight pathogen *P. infestans*. These cultivars are listed in Table 1 along with their resistance background and cultivar maturity. The trial was laid out in a randomized block design with three replications. The spacing was maintained at 60 cm between rows and 25 cm between plants, with a total of 40 plants per row and 2 rows per plot, and a row length of 10 m. Before planting, the field was fertilized with the organic fertilizer Black Pearl (BIOCAT G), norm 300 kg ha^−1^ (30 kg N ha^−1^, 15 K kg ha^−1^, 25.5 S kg ha^−1^). No pesticides were used according to EU regulations on organic production (Council Regulation N0. 843/2007).

### 2.2. Volatile Collection and Gas Chromatography-Mass Spectrometry Analyses

Volatile organic compounds were collected from the leaves of nine potato cultivars under late-blight-free conditions on 16 August 2018 under natural temperature conditions in full sunlight (photosynthetic photon flux density of 1000–1400 μmol m^−2^ s^−1^). A total of 27 plants (three per cultivar representing biological replicates) were used for volatile sample collection. One mature healthy leaf per plant was carefully inserted in a Tedlar bag and closed around the petiole [43]. To trap all volatiles in the C3–C17 range, stainless steel cartridges filled with three different carbon-based adsorbents Carbotrap C 20/40 mesh, Carbopack B 40/60 mesh, and Carbotrap X 20/40 adsorbents (Supelco, Bellefonte, PA, USA) were used for VOC collection [44]. Two air sample pumps (210–1003 MTX, SKC Inc., Houston, TX, USA) were affixed to the air outlet and inlet port of the Tedlar bag. Initially, the air was pumped through a VOC collection cartridge for 10 min to minimize the effect of VOC emissions from the surrounding environment. Subsequently, a second air pump was activated to collect leaf VOCs at a constant flow rate of 0.2 L min^−1^ for 20 min on adsorbent cartridges. The method was optimized using in-vivo-grown potato plants before conducting the field experiment. The volatile samples in the cartridges were desorbed with a Shimadzu TD-20 automated cartridge desorber and analyzed with a Shimadzu 2010 Plus gas chromatography–mass spectrometer (GC–MS; Shimadzu, Kyoto, Japan) with a Zebron ZB-624 fused silica capillary column (0.32 mm i.d., 60 m length, 1.8 μm film thickness, Phenomenex, Torrance, CA, USA) according to the protocol described in [44]. Each compound was identified based on mass spectra of pure standards (Sigma-Aldrich, St. Louis, MO, USA) and NIST 14 spectral library with a 95% probability (National Institute of Standards and Technology), and the peaks were integrated with the open-access program OpenChrom ver. 1.2.0 (Alder) [45]. The emission rates were calculated as in [43].

### 2.3. Estimation of Leaf Dry Mass Per Unit Area

After VOC sampling, the leaves were harvested, and the fresh mass and area were estimated immediately. For the leaf area, the leaves were photographed, and the area was calculated with ImageJ software (National Institute of Health, Bethesda, MD, USA). The leaves were dried at 70 °C for 48 h in a drying oven, and their dry mass was determined. Leaf dry mass per unit area (LMA) was calculated [43]. Variation in leaf dry mass per unit area was small among the cultivars, from 18.1 ± 2.3 g m^−2^ in Anti to 24.4 ± 4.6 g m^−2^ in Kuras (Table 1), and thus, the differences in the emission rates among the cultivars are similar (*p* > 0.05) when the emissions were expressed per unit dry mass (Table 1).

### 2.4. Statistical Analyses

Three biological replicates were used for all quantifications, and the data of the study’s findings were expressed as the average of those replicates. Data for the VOCs and LMA were tested for normality (Shapiro–Wilk test) and equality of variances (Levene’s test) before analysis. Ln-transformation was applied when necessary to improve the distribution of data and/or variances. One-way ANOVA was used to test the differences among the cultivars for VOCs and LMA. The Kruskal–Wallis test was also used. Principal component analysis (PCA) and hierarchical cluster analysis were applied to analyze the differentiation of genotypes according to the VOC profiles. PCA can detect the data structure and determine the relationships between samples (in this case potato cultivars) and original variables (volatile profiles). Here, new variables, called principal components, are calculated as a linear combination of the original variables such that the first component takes up higher amounts of the variances of the original variables [46]. With PCA, we can reduce the dimension of the VOC profiles to a linear combination of variables (principal components) and also group genotypes based on the studied traits [39,40,46,47]. The technique has a wide domain of applications including use in VOC studies [39,40,46,47,48,49]. The output of the analysis is expressed in terms of principal components (PC), their % variance, and loading of the variables. All statistical tests were considered significant at *p* < 0.05. The R version 4.2.0 statistical program [50] was used for all statistical analyses and visualizations.

## 3. Results

### 3.1. Constitutive VOC Emission Rates in Leaves of Potato Cultivars with Different Late Blight Resistance Backgrounds

**Figure 1:**
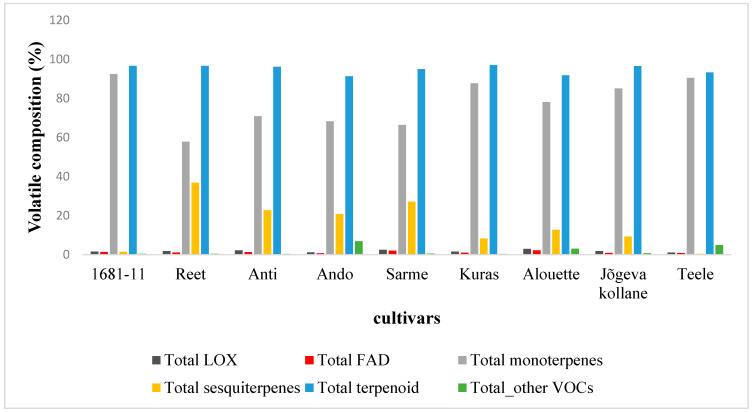
Volatile profile composition (relative proportion) across nine potato cultivars with varying resistance levels (see Table 1). FAD: long-chained saturated-fatty-acid-derived compounds; LOX: classic green leaf volatiles; terpenoid (monoterpenes, sesquiterpenes, and isoprene); others: sum of all other emitted VOC.

Forty-six VOCs (Table 2) were identified in constitutive emission blends of nine potato cultivars in the field. The compounds emitted were lipoxygenase (LOX) pathway volatiles (a classic green leaf volatile), long-chained saturated-fatty-acid-derived (FAD) compounds, terpenoids (isoprene, monoterpene, and sesquiterpene), geranylgeranyl diphosphate pathway (GGDP) volatiles, and acetaldehyde. Most of the detected volatiles were terpenoids (monoterpenes and sesquiterpenes) and depending on the cultivar, they represented between 57.8–92.5% for monoterpenes and between 0.5–36.9% for sesquiterpenes of the total emissions (Table 2, Figure 1). The variation in the total VOC emissions rate was moderate, with the highest average ± SE value of 1916 ± 765 pmol m^−2^ s^−1^ in Kuras and the lowest value of 888 ± 97.6 pmol m^−2^s^−1^ in Teele (Table 2). There were no significant differences in the total emission rates among the cultivars (Table 2).

However, the share of the VOCs among different compound classes varied among the cultivars, implying unique cultivar-specific VOC blends. Significant differences (*p* < 0.05) in monoterpene emissions among the cultivars were only observed in tricyclene and α-terpinene, and overall, there were no significant differences in total monoterpene emissions among the cultivars. For the sesquiterpenes, the emission rates of most of the individual volatiles and total emission rates differed among the cultivars (Table 2). The variation in the emission rates of terpenoids was moderate with the lowest value of 829 ± 73.1 pmol m^−2^ s^−1^ in Teele, while Kuras had the highest value of 1860 ± 753 pmol m^−2^ s^−1^, with a variation of between 91.3 to 97.1% of the total emission rate among the cultivars (Figure 1). Among the monoterpenes, α-pinene (55.8–61.6%), Δ^3^-carene (17.5–20.3%), β-pinene (5.8–7.2%), and limonene (7.4–9.0%) dominated the monoterpene emissions (Table 2). All of the cultivars released the same monoterpenes except the cultivar Reet which also emitted α-terpinene (Table 2). All of the cultivars emitted three sesquiterpenes, (*E*)-β-caryophyllene, longifolene, and α-copaene, that together accounted for 14.2–76.7% and 0.4–22.2% of the total sesquiterpenes and total VOC emissions, respectively (Table 2). All of the cultivars, except Teele, also emitted (*E*)-β-farnesene and β-elemene (Table 2). The emissions of the other sesquiterpenes, β-gurjunene, α-gurjunene, α-ylangene, δ-cadinene, β-cedrene, α-cubebene, α-humulene, β-santalene, α-caryophyllene, β-bergamotene, γ-muurolene, α-muurolene, γ-cadinene, etc., were highly cultivar dependent (Table 2).

The emission rate of long-chained saturated-fatty-acid-derived (FAD) compounds varied from 6.8 ± 5.5 pmol m^−2^ s^−1^ in Teele to 39.1 ± 5.0 pmol m^−2^ s^−1^ in Alouette (Table 2). The emission rate of classic green leaf volatiles (also called LOXs; hexanal and hexanol) varied from 9.6 ± 7.5 pmol m^−2^ s^−1^ in Teele to 53.2 ± 16.7 pmol m^−2^ s^−1^ in Alouette. In total, the LOXs and FAD emission rates constituted between 1.1–3.0%, and 0.7–2.2% of the total VOC emissions, respectively (Figure 1). In the VOC emissions, hexanal dominated the LOXs. All of the cultivars emitted the same set of LOXs and FAD compounds (Table 2). The total emissions of geranylgeranyl diphosphate (GGDP-pathway volatile) 6-methyl-5-hepten-2-one (carotenoid breakdown products) were low overall (4.7 ± 0.4 pmol m^−2^ s^−1^–10.2 ± 1.1 pmol m^−2^ s^−1^), with an average of 6.6 ± 0.6 pmol m^−2^ s^−1^, and there were no significant differences among the cultivars (Table 2). The cultivars did not differ significantly for isoprene emissions.

### 3.2. Cultivar Differences in the Blend of Emitted Volatiles

Principal component analysis (PCA) was used to determine which characteristics contributed to the overall diversity among the cultivars. The forty-six VOC variables were reduced to only seven principal components (PCs), having eigenvalues greater than 1 (Table S1). For clarity of presentation, only four PCs are shown (Table 3). The four principal components (PCs) had a cumulative variance level of 71.2%, representing PC1 (23.3%), PC2 (19.5%), PC3 (15.5%), and PC4 (13.0%). The first two PCs accounted for 42.7% of the total variation among the cultivars. The compounds mainly associated with the first component (PC1) were linked to monoterpenes that dominated PC1 and could thus be termed the monoterpene component. The second component (PC2) was strongly associated with LOXs (classic green leaf volatile) and long-chained saturated-fatty-acid-derived (FAD) compounds, which accounted for 19.5% of the total variation among the cultivars. Sesquiterpenes were widely distributed across the PC components.

**Figure 2:**
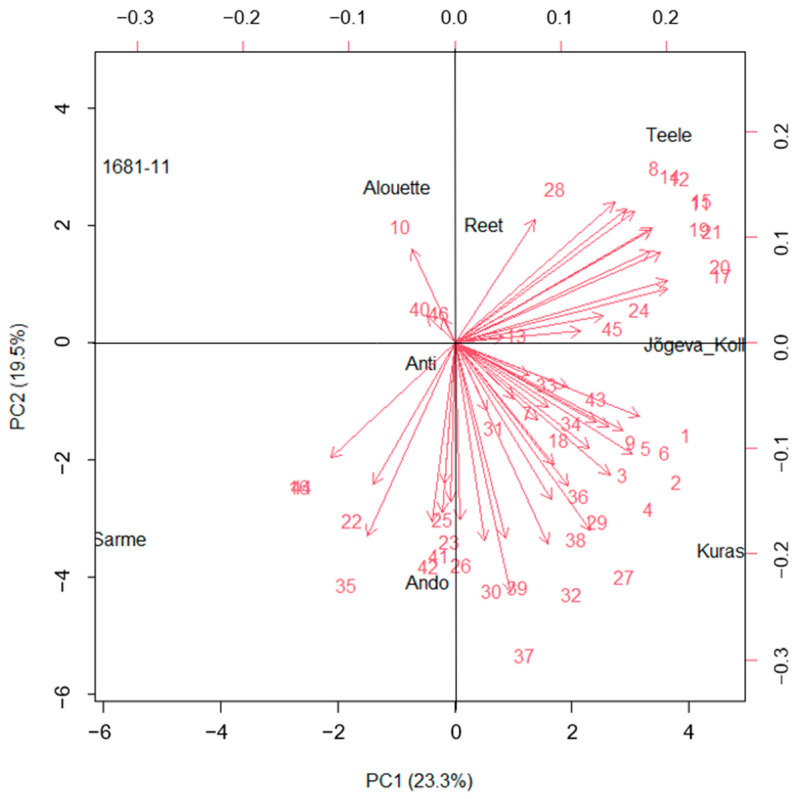
Biplot of the first two principal components of the emitted VOCs showing the distribution of different potato cultivars. Refer to Table 2 for the numbers assigned to each VOC.

Thus, monoterpenes on the one hand, and FAD compounds (including LOXs) which mainly constitute the first and second principal components, respectively, accounted for greater variation among the cultivars. Overall, the results revealed the importance of LOXs, FAD compounds, and monoterpenes in the variation among the potato cultivars based on the VOC profiles. The clustering pattern according to the principal components biplot for all the VOCs combined tends to delineate the cultivars according to their resistance categories (Figure 2). The highly resistant cultivars Ando, Anti, and Sarme were grouped into the same quadrant, while Alouette and Kuras were in a separate quadrant. Thus, the biplot using the VOC profile can also identify sub-groups within resistant cultivars. The low-resistance cultivars Reet, Teele, and Jõgeva Kollane were grouped into the same quadrant, separate from the highly resistant cultivars. Based on the PC axis, the highly resistant cultivars Sarme, Ando, Kuras, and Anti can also be observed to be clustered on the negative side of the PC2 axis, while the low-resistance cultivars were on the positive side of the PC2 axis, except Alouette, the high resistance cultivar that was in the same cluster as 1681-11. This disparity can occur as the biplot can only capture two components, and thus, this explained not all but some portion (42.8%) of the variation.

**Figure 3:**
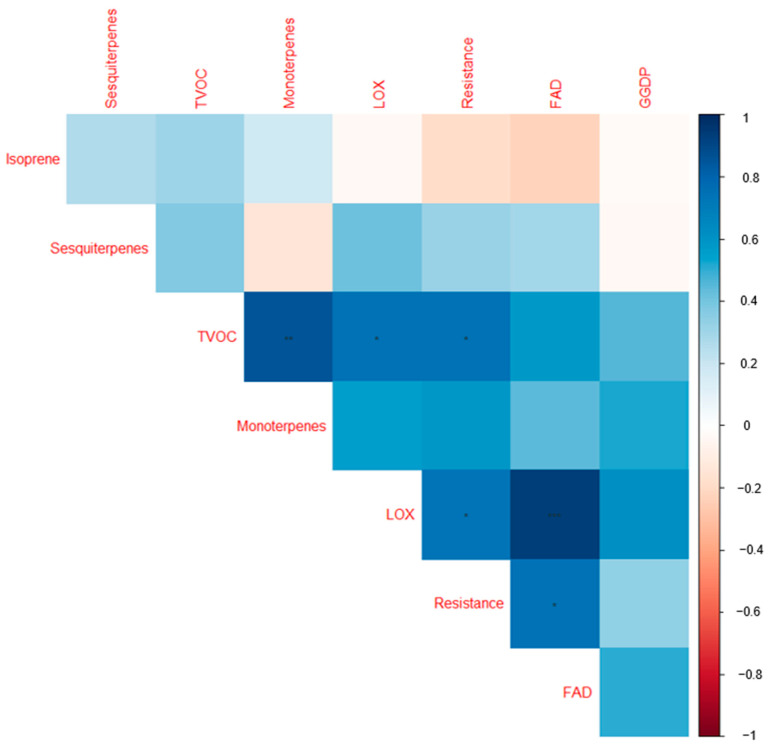
Correlogram visualizing correlations among the volatile organic compound groups and the cultivar resistance scores for the nine cultivars studied. The colors are proportional to the correlation coefficients. The positive and negative correlations are indicated in blue and red. A greater correlation coefficient is reflected by a color of higher intensity. * = significant at 0.05; ** = significant at 0.01; *** = significant at 0.001. FAD: long-chained saturated-fatty-acid-derived compounds; LOX: classic green leaf volatiles; geranylgeranyl diphosphate (GGDP): 6-methyl-5-hepten-2-one; TVOC: total volatile organic compound emissions; resistance: late blight resistance scores in the nine cultivars studied (Table 1). Table 2 provides the full list of compounds observed in the current study.

A separate clustering based on each individual VOC group (Figure S1): FAD, including LOXs (Figure S1a), monoterpenes (Figure S1b), and sesquiterpenes (Figure S1c), did not delineate the cultivars according to the resistance categories. In the biplot (Figure S1a), the LOXs tend to be more closely correlated, likewise for the FAD compounds. However, the biplot based on the terpenoids (monoterpenes, sesquiterpenes, and isoprene combined) shows some extent of clustering of the cultivars according to their resistance categories (Figure S1d). The low-resistance cultivars Reet, Teele, Jõgeva Kollane, and 1681-11 were all clustered on the negative axis of PC2, where Reet, Teele, and Jõgeva Kollane were in a separate quadrant. On the other hand, the highly resistant cultivars Ando, Sarme, Anti, and Kuras were grouped on the positive PC2 axis. The biplot based on the terpenoids accounted for 54.6% of the variation. As shown in the heatmap (Figure 3), there was a significant correlation between cultivar resistance and total VOC emissions.

**Figure 4:**
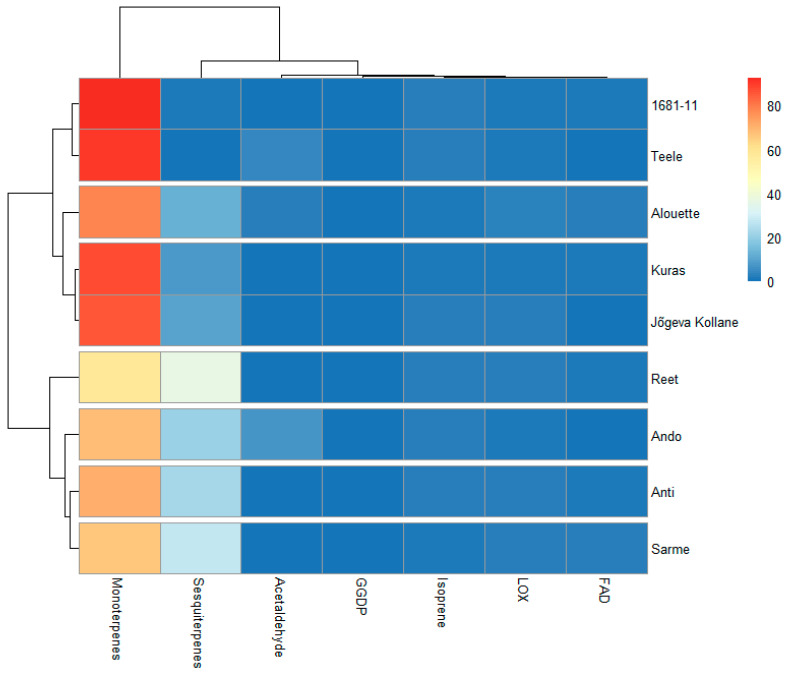
Heatmap with a dendrogram illustrating the groupings of the studied potato cultivars with different late blight resistance backgrounds (Table 1 for the studied cultivars) based on the quantities of the plant secondary metabolites (% relative to the total VOCs) emitted from the leaves. The colors indicate relative amounts of the compounds. Blue and red represent the lowest and highest levels, respectively. FAD: long-chained saturated-fatty-acid-derived compounds; LOX: classic green leaf volatiles; geranylgeranyl diphosphate (GGDP): 6-methyl-5-hepten-2-one.

Individual pathway groups also tend to exist separately and are uncorrelated with other pathway groups (Figure 3). However, the FAD and LOXs were positively correlated (*p* < 0.001). In a separate analysis, resistance also scales positively with terpenoids (isoprene, monoterpenes, and sesquiterpenes combined) (*p* < 0.05). A heatmap was constructed to visualize the share of the VOC classes to the total VOC emissions (% relative to the total VOC emissions) among the cultivars (Figure 4).

**Figure 5:**
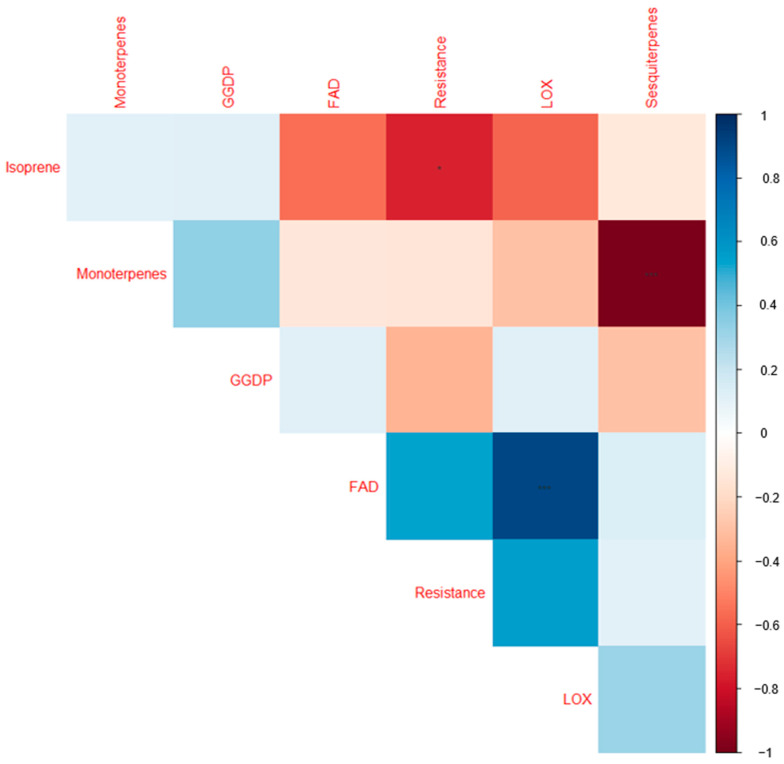
Correlogram visualizing the correlations between the share of secondary metabolites (% relative to the total VOCs) and cultivar resistance. The colors are proportional to the correlation coefficients. The positive and negative correlations are indicated by blue and red, respectively. A greater correlation coefficient is reflected by a color of higher intensity. * = significant at 0.05; *** = significant at 0.001. FAD: long-chained saturated-fatty-acid-derived compounds; LOX: classic green leaf volatiles; geranylgeranyl diphosphate (GGDP): 6-methyl-5-hepten-2-one.

Two major clusters that did not correspond to the highly resistant and low-resistance cultivars can be noticed. However, sub-clusters containing highly resistant cultivars such as Ando, Anti, and Sarme that were grouped together, as well as the low-resistance cultivars 1681-11 and Teele that also form a sub-cluster, can be noticed, while the cultivar Reet tend to form a singleton. On the other hand, Jõgeva kollane tended to share some unique profiles with the highly resistant cultivars Alouette and Kuras. Except for isoprene, there were no significant correlations between the shares of the VOCs classes with resistance (Figure 5).

## 4. Discussion

### 4.1. Diversity of Volatile Profiles Compared with Other Commercial Potato Genotypes (Solanum tuberosum) and Solanum spp.

In the temperate zone, potato (*Solanum tuberosum*) is among the commonly cultivated commercial plants [46] and an important food source in many parts of the world [51]. It is an important crop in many countries, including Estonia. At the constitutive level, plants including *Solanum* spp. emit a huge number of volatile metabolites [20,46,51], and most of the VOCs have defensive and attractive roles [23,25,51]. VOC fingerprints tend to be unique among different plants and varieties [22]. However, the results obtained here are consistent with previous research on scattered varieties of *Solanum* spp., as the identified compounds in these studies overlapped heavily with those identified in other studies of VOCs in the foliage of *Solanum* spp. [22,46,52,53]. Sesquiterpenes and monoterpenes were the dominant volatiles, as is common in potatoes [22,51]. Similar to other studies in *Solanum tuberosum* foliage [46], the composition of sesquiterpenes was cultivar specific. As observed in other studies, β-caryophyllene common to all cultivars was among the dominant sesquiterpenes in the foliage of the studied cultivars and also constitutes a quantitative marker for differentiation of potato cultivars [46]. Similarly, α-cubebene and δ-cadinene were also present in small quantities. These qualitative and quantitative differences in sesquiterpenes can constitute a marker for varietal classification and characterization [46].

### 4.2. Quantitative and Qualitative Differences in Constitutive VOC Emissions among the Cultivars

There is evidence that different plant cultivars of given crop species might produce different suites of volatiles [20,39,41,47,48]. The inherent variability of constitutive VOC emissions among plant genotypes has been studied and differences in specific VOC abundance between resistant and susceptible genotypes has been observed [24,40]. Constitutive VOC emissions have been involved in defense against pathogen attacks without the time delay that is involved in the production of inducible defenses [54]. This encouraging evidence inspired us to characterize constitutive VOC emissions among potato cultivars with varying resistance to *P. infestans*.

We observed that the various volatile pathways largely operate independently and are uncorrelated with other pathway groups. This may be due to the independent regulation of different metabolic pathways in plants that could arise from specific transcription factors that control the expression of the genes involved in a specific pathway or as a result of different regulations by the key pathway flux-controlling enzymes [49]. Metabolites formed via a common pathway or originating from a common precursor tend to have a close relationship with each other [38,49]. Strong correlations among the VOCs indicated as clusters for the factor loadings of the PCA may mostly reflect common biosynthetic pathways among the volatiles concerned [38]. Cultivars differ in VOC emission composition, and significant differences were observed for total sesquiterpenes emissions among the cultivars. Differences in emission rates also exist among some of the individual compounds, with higher emissions in some of the highly resistant cultivars. Differences in specific VOCs that belong to aldehyde, monoterpene, and sesquiterpenes were also observed in more tolerant (to huanglongbing) citrus varieties [40]. Classic LOXs were emitted in low quantities similarly in all cultivars and this might reflect mild chronic stress in the field that might often remain unnoticed. In tomato foliage, the concentration of classic green volatiles was relatively low. However, during damage, the concentration increased drastically [53]. These LOX products are produced by the oxidation of lipid components of damaged cells in green vegetables to form lipid hydroperoxides, which rapidly break down to form many compounds, including C_6_ aldehydes and alcohols [23,55]. Cultivar resistance scaled positively with total LOX products and FAD compound emissions and may be related to their inhibitory properties against pathogens. Aldehydes from LOX products are highly reactive and can diffuse from sites of production to extracellular targets, due to longevity [56], and they have inhibitory properties against pathogens [57,58,59,60,61,62,63,64].

The emitted VOCs were dominated by terpenoids, (monoterpenes or sesquiterpenes). The constitutive emission of GDP-pathway compounds is common in several plant species [65], including *Solanum* spp. such as tomato (*S. lycopersicum*) [20], potato [22,46], and citrus varieties [39], etc. Terpenes are important in biological processes related to plant defense against biotic and abiotic stresses [66] and constitute the largest class of plant compounds among the plant secondary metabolites. They can provide defense against pathogens whether in constitutive or induced emission [54,67]. Terpenoids from the root extract of different cultivars of *Chrysanthemum Morifolium* have shown significant inhibition in the growth of fungal pathogens, and variation exists in terpenoid emissions and inhibition ability among the cultivars [68]. Interestingly, we observed that cultivar resistance scales positively with total terpenoid emissions. In *Chrysanthemum Morifolium*, the root extracts from cultivars with the highest terpenoid emissions did not always correspond to the highest inhibitory effect [68]. On the other hand, in *Pinus thunbergii,* the expression of terpene synthesis genes was higher in the resistant (to pine wilt disease) genotype compared to the susceptible one, and there was significantly more induced terpenoids in the resistant genotype [69]. It is tempting to speculate that the individual terpenoids or their blend ratios may play a greater role in pathogen inhibition, rather than the total terpenoid emissions. However, if we consider the indirect effect such that the induction of terpenoids triggers defense signaling pathways, it makes sense to relate increased terpenoids with higher resistance in addition to their direct inhibitory effect. Moreover, volatile terpenes are known to constitute part of the direct or indirect plant defense against pathogens [70,71]. Under disease conditions, grapevines with a lower infection rate (for *Plasmopara viticola*) have also been shown to emit a relatively higher quantity of total terpenes [70]. Striking differences were found in the composition of the sesquiterpenes among the potato cultivars. Only three sesquiterpenes, (*E*)-β-caryophyllene, longifolene, and α-copaene, were emitted by all of the cultivars, irrespective of the genetic background. α-cubebene, α-humulene, and α-bergamotene were present in the highly resistant cultivars Alouette, Kuras, and Sarme but absent in the low-resistance cultivars Teele and 1681-11. Likewise, γ-muurolene was present in Alouette and Kuras but absent in the low-resistance cultivars Teele, Reet, and 1681-11. The qualitative and quantitative differences in sesquiterpenes emissions among the cultivars may reflect the presence of specific enzymes and the activities of the cytosolic mevalonate pathway that might have resulted in the differences in the VOC fingerprint [49]. This cultivar specificity in sesquiterpene emissions is not only common to potatoes but has also been observed among Tristeza-virus-tolerant and susceptible citrus varieties [39] and even in the roots of *Chrysanthemum morifolium* cultivars [68]. Sesquiterpenes are involved in defense response to pathogens through JA signaling [72]. Higher emissions of some of these individual sesquiterpenes may have a direct effect on pathogens even before the activation of inducible defenses [54]. Constitutive expression of the sesquiterpene (*E*)-β-caryophyllene reduces *Pseudomonas syringae* infection in *Arabidopsis thaliana*, and when muted, the plant experiences increased growth of the pathogen [54].

The observed qualitative differences indicate that specific VOC fingerprints may characterize different potato cultivars even according to resistance categories against *P. infestans*. Constitutive monoterpene synthesis occurs in plastids where the terminal enzymes and monoterpene synthases are located [73,74]. These constitutive monoterpene emissions could arise from specialized storage tissues [75]. The synthesis of isoprene and monoterpene depends on the same precursor dimethylallyl pyrophosphate (DMADP) and is enzymatically synthesized via the MEP/DOXP pathway in plastids [74,76]. In the current study, isoprene emissions were relatively low and was not correlated with monoterpene emissions. Isoprene is emitted from many plant species at trace levels and these emissions could be non-enzymatic or reflect the mixed substrate activity of some terpene synthases [77]. Fatty-acid-derived compounds, including LOXs contributed to a greater percentage of variation among the cultivars, followed by monoterpenes indicating that the LOX and MEP/DOXP pathways that are involved in the production of these compounds may behave differently among the cultivars. The compounds more positively correlated with the first component for the total VOC emissions were mainly linked to monoterpenes as also observed among citrus varieties with varying resistance to Tristeza virus [39]. Moreover, higher emissions of some of the individual monoterpenes such as limonene, α-pinene, β-pinene, and Δ^3^-carene in all the cultivars may suggest a possible role of these volatiles as a direct defense. These compounds are well known for their antimicrobial activities [27,78,79,80,81,82,83,84]. α-pinene was only detected in moderately and highly resistant *Pinus massoniana* to pine wilt disease, and α-pinene and β-pinene were among the main VOCs in the emission profile [85]. Higher emissions of limonene, and α -pinene have been observed in citrus varieties tolerant to the Tristeza virus [39].

### 4.3. Are Constitutive Volatile Organic Compounds Emissions Associated with Cultivar Resistance to Potato Late Blight?

Volatile organic compounds (VOCs) emitted by plants can reveal information about plant defense processes and are known to have a strong genetic component and are often related to multiple plant phenotypic traits [25,86]. Hence, they can be a good phenotyping marker [86]. Estimation of a plant’s chemical phenotype by monitoring its VOC profile is a fast and non-invasive method, and the key question is whether the chemical phenotype is associated with plant disease resistance and/or stress tolerance. The study of constitutive plant secondary metabolite profiles of late-blight-susceptible and tolerant potato cultivars could provide insight into the potential role of key metabolites in late blight resistance [25,39]. Interestingly, cultivar resistance scales positively with total VOC emissions. Thus, constitutive VOC emissions in potato genotypes may show promise as a phenotypic marker for late blight resistance. The relatively higher emission rate and variation in some individual VOC compounds among the cultivar could indicate their direct role in defense response, even before inducible defenses are activated [54]. Constitutive plant volatiles have shown promise in the differentiation of citrus varieties for resistance to Huanglongbing [40], where tolerant cultivars contain a higher amount of total VOCs. VOC emissions have also shown potential in characterizing pea cultivars based on resistance categories [86]. Under disease conditions, higher VOC emissions in grapevine correlate with lower disease severity [70]. However, induced emissions under pathogen attack might be differently elicited among potato cultivars. Hence, future work should look at induced emissions. The results of the study may help in the understanding of the mechanism of potato tolerance against late blight disease and provide useful information on their secondary metabolism. This will also open the possibility to exploit the phenomenon of pathogen-induced volatile emissions for the control of late blight by breeding varieties with enhanced induced VOC emissions. Apart from leaves, oospores can survive in the soil until the next potato crop and infect potato plants through the root. Thus, the root VOCs can play an important role in disease resistance [68], acting either directly by repelling biotic stressors or through molecular recognition and signaling leading to effective resistance. To fill these gaps, our future studies will investigate the intraspecific variability of the below-ground emissions of VOCs among potato cultivars under both stress and stress-free condition related to *P. infestans* to know whether VOCs from above and below ground are partially interconnected. This will improve our understanding of the role of VOCs in plant–pathogen interaction. Our future work will also address issues concerning the number of cultivars with a larger study to further this research.

## 5. Conclusions and Outlook

The complex mixture of constitutive volatile emissions in potato leaves spans a large number of volatile products, including LOX products, FAD compounds, monoterpenes, sesquiterpenes, GGDP pathway products, and oxygenated volatile organic compounds. The emission blend of the cultivars was rich in volatiles that have been known to have anti-microbial activities. This study identifies the specific characteristics of the chemical composition of the volatile compounds for nine potato cultivars with different levels of late blight resistance and suggests that constitutive VOC emissions can be a promising marker to phenotype potato cultivars for late blight resistance. The findings could also contribute to a better understanding of the diversity of the chemical profiles of *Solanum tuberosum* foliage.

## Figures and Tables

**Table 1:** List of potato cultivars studied, cultivar maturity, foliar resistance to the late blight pathogen, leaf dry mass per unit area, and total emission rate per unit dry mass.

Cultivar *	Maturity	Foliar Resistance to Late Blight (*P. infestans*)	Leaf Dry Mass Per Unit Area (g m^−2^) ^#^	Total Emission Rate Per Unit Dry Mass (pmol g^−1^ s^−1^)
Alouette	medium	9	20.7 ± 0.3	112.9 ± 75.3
Sarme	late	8	19.2 ± 1.9	91.4 ± 24.9
Kuras	late	8	24.4 ± 4.6	88.3 ± 31.2
Ando	late	7	20.3 ± 0.4	93.9 ± 42.4
Anti	late	7	18.1 ± 2.3	121.2 ± 54.6
Jõgeva kollane	late	5	22.6 ± 1.1	76.6 ± 15.3
Teele	medium	5	18.4 ± 1.8	49 ± 2.7
1681-11	medium	4	22.0 ± 0.4	55.2 ± 25.4
Reet	medium	4	20.3 ± 0.7	66.7 ± 17.2
*p*-value	–	–	0.284	0.675

* 1681-11 is a promising breeding line for organic farming. The resistance to *Phytophthora infestans* is a rank value defined as 1—very low, 2—very low to low, 3—low, 4—low to medium, 5—medium, 6—medium to high, 7—high, 8—high to very high, and 9—very high (European Cultivated Potato Database https://www.europotato.org/; accessed on 5 May 2022), and the breeder information is from the Centre of Estonian Rural Research and Knowledge (METK). *p*-value: Significance of variation among the cultivars. ^#^ The leaf dry mass per unit area was estimated for three replicate leaves. Data are given as the averages ± SE. The average dry mass per unit area was 20.5 ± 0.6 g m^−2^, and there were no significant differences among the cultivars (*p* > 0.05).

**Table 2:** Mean (±SE, n = 3) emission rates (pmol m^−2^s^−1^) of volatile organic compounds (VOCs) among the nine potato cultivars with different late blight resistance backgrounds.

	Compounds	Cultivar									
	Lipoxygenase Pathways (LOX) Volatiles	1681-11	Reet	Anti	Ando	Sarme	Kuras	Alouette	Jõgeva Kollane	Teele	*p*-Value
1	1-hexanol	1.7 ± 0.5	1.8 ± 0.4	3.1 ± 1.0	1.7 ± 0.5	2.2 ± 1.7	2.6 ± 0.5	4.3 ± 0.8	2.2 ± 0.9	1.0 ± 1.0	0.366
2	hexanal	17.5 ± 5.6	19.5 ± 5.2	38.9 ± 22.9	17.2 ± 4.7	33.2 ± 19.6	27.8 ± 10.4	48.9 ± 16.0	26.1 ± 7.6	8.6 ± 8.6	0.429
	Total LOX compounds	19.2 ± 6.1	21.3 ± 5.6	42.0 ± 24.0	18.9 ± 5.1	35.4 ± 21.3	30.4 ± 11.0	53.2 ± 16.7	28.3 ± 8.2	9.6 ± 7.5	0.422
	**Long-chained saturated-fatty-acid-derived (FAD) compounds**										
3	decanal	2.6 ± 1.0	2.4 ± 1.2	4.1 ± 4.4	2.0 ± 0.2	6.1 ± 1.8	3.4 ± 0.8	8.5 ± 1.0	2.3 ± 1.9	1.1 ± 1.0	0.281
4	heptanal	2.4 ± 0.7	2.0 ± 0.5	3.8 ± 1.7	2.2 ± 1.1	4.8 ± 0.4	2.9 ± 0.6	5.2 ± 0.5	2.4 ± 0.9	1.1 ± 1.2	0.137
5	nonanal	8.1 ± 3.1	5.0 ± 2.1	10 ± 6.1	3.6 ± 0.3	11.1 ± 3.1	7.8 ± 2.5	16.5 ± 2.7	5.3 ± 5.7	2.5 ± 2.1	0.415
6	octanal	3.9 ± 1.1	3.0 ± 0.8	5.9 ± 2.6	3.2 ± 0.9	6.4 ± 1.5	5.0 ± 1.6	8.9 ± 1.0	3.9 ± 1.8	2.1 ± 2.3	0.232
	Total FAD compounds	17 ± 5.4	12.4 ± 4.6	23.8 ± 14.8	11.0 ± 2.4	28.4 ± 6.3	19.1 ± 5.4	39.1 ± 5.0	13.9 ± 10.3	6.8 ± 5.5	0.292
7	**Isoprene**	32.8 ± 1.8	22.7 ± 7.5	46.7 ± 25.0	37.2 ± 15.2	21.8 ± 4.9	21.8 ± 6.9	16.8 ± 1.2	32.9 ± 5.6	20.5 ± 3.3	0.268
	**Monoterpenes**										
8	camphene	16.4 ± 2.5	10.4 ± 1.0	18.9 ± 7.0	19.5 ± 8.1	12.9 ± 7.2	26.7 ± 9.8	17.4 ± 5.1	19.6 ± 7.3	13.4 ± 0.8	0.578
9	camphor	2.3 ± 1.1	1.4 ± 0.4	2.8 ± 1.4	1.8 ± 0.2	1.1 ± 1.3	1.3 ± 1.3	2.1 ± 0.8	1.6 ± 0.6	1.0 ± 0.3	0.385
10	eucalyptol	1.3 ± 0.9	1.0 ± 0.2	1.3 ± 0.4	1.0 ± 0.3	1.5 ± 0.7	1.3 ± 0.5	1.3 ± 0.3	1.3 ± 0.1	2.1 ± 0.7	0.861
11	limonene	94.3 ± 18.2	52.3 ± 3.0	112.9 ± 44.1	98.4 ± 38.0	70.6 ± 65.7	142.8 ± 59.5	123.9 ± 40.8	116.4 ± 15.1	63.9 ± 13.8	0.662
12	*p*-cymene	29.9 ± 4.5	16.7 ± 0.6	34.6 ± 11.6	33.2 ± 26.8	26.8 ± 20.0	45.4 ± 17.0	33.9 ± 12.0	36.7 ± 9.6	24.9 ± 2.1	0.693
13	tricyclene	3.3 ± 0.5	12 ± 11.4	14.2 ± 5.1	29.1 ± 21.9	2.5 ± 1.0	22.9 ± 12.4	4.2 ± 1.3	7.6 ± 2.3	3.2 ± 0.1	0.005
14	α-fenchene	5.7 ± 0.8	2.8 ± 0.2	6.4 ± 1.7	5.7 ± 2.3	5.0 ± 2.6	8.5 ± 2.7	6.0 ± 2.0	7.7 ± 1.0	4.2 ± 0.2	0.387
15	α-pinene	677.6 ± 119.7	382.4 ± 2.5	771 ± 270.6	625.3 ± 232.7	572.1 ± 344.1	982.5 ± 357.8	807.6 ± 295.8	757.4 ± 133.9	495.1 ± 24.5	0.620
16	α-terpinene	nd	1.9 ± 0.3	nd	nd	nd	nd	nd	nd	nd	0.001
17	α-thujene	1.7 ± 0.3	1.0 ± 0.1	1.9 ± 0.7	1.4 ± 0.6	1.5 ± 1.0	2.0 ± 0.8	2.1 ± 0.8	1.7 ± 0.2	1.0 ± 0.1	0.576
18	β-myrcene	6.8 ± 2.1	10.0 ± 6.4	13.2 ± 6.5	21.2 ± 15.2	7.6 ± 5.2	11.1 ± 6.6	11.8 ± 5.8	7.5 ± 0.1	3.8 ± 1.1	0.303
19	β-phellandrene	6.8 ± 2.0	3.6 ± 0.2	7.1 ± 4.5	6.8 ± 3.3	5.1 ± 5.5	9.5 ± 5.7	9.9 ± 4.1	6.5 ± 0.8	4.2 ± 1.0	0.778
20	β-pinene	77.3 ± 17.3	43.4 ± 3.8	96.1 ± 234.9	67.9 ± 27.6	64.4 ± 49.2	107.7 ± 43.0	97.4 ± 36.5	79.2 ± 4.5	46.5 ± 9.5	0.639
21	Δ^3^-carene	221.2 ± 46.6	126.6 ± 3.4	261.6 ± 100.6	208.9 ± 74.3	178.8 ± 139.0	317.8 ± 135.8	283.7 ± 108.6	247.6 ± 35.7	140.6 ± 24.3	0.664
	Total monoterpenes	1144.6 ± 215.4	665.5 ± 22.8	1342 ± 485.8	1120.2 ± 433.0	949.9 ± 639.2	1679.5 ± 650.7	1401.3 ± 512.6	1290.8 ± 207.0	803.9 ± 75.1	0.465
	**Sesquiterpenes**										
22	(E)-β caryophyllene	6.8 ± 4.3	242.6 ± 177.7	53.5 ± 53.0	111.4 ± 76.6	33.6 ± 33.2	50.2 ± 46.7	43.0 ± 35.9	26.7 ± 6.8	1.0 ± 1.0	0.000
23	(*E*)-β -farnesene	2.2 ± 0.9	12.5 ± 7.3	11.0 ± 10.4	44.0 ± 34.7	63.0 ± 17.4	11.0 ± 7.4	5.1 ± 1.7	4.8 ± 0.8	nd	0.016
24	longifolene	2.6 ± 1.9	1.9 ± 0.6	3.1 ± 1.8	1.7 ± 1.1	1.9 ± 1.3	3.2 ± 1.1	2.1 ± 0.5	1.9 ± 0.5	1.3 ± 0.8	0.737
25	thujopsene	nd	nd	nd	nd	13.1 ± 4.4	nd	nd	nd	nd	0.029
26	valencene	nd	3.4 ± 1.2	4.7 ± 1.0	nd	nd	nd	nd	nd	nd	0.001
27	α -bergamotene	nd	15.4 ± 5.2	65.0 ± 21.7	28.1 ± 23.9	32.7 ± 12.4	7.7 ± 7.7	54.4 ± 42.1	6.1 ± 1.5	nd	0.029
28	α- caryophyllene	nd	nd	nd	nd	nd	10.4 ± 3.5	nd	nd	nd	0.000
29	α-copaene	3.9 ± 1.0	10.6 ± 3.9	68.6 ± 47.9	18.8 ± 14.5	19.6 ± 6.5	15.9 ± 4.6	5.9 ± 1.1	20.8 ± 6.9	1 ± 1	0.026
30	α-cubebene	nd	5.0 ± 3.5	6.0 ± 2.7	3.5 ± 2.8	11.6 ± 2.5	4.2 ± 1.6	1.1 ± 0.9	4.5 ± 1.8	nd	0.018
31	α-gurjunene	nd	11.7 ± 8.5	8.6 ± 2.6	19.8 ± 12.1	nd	nd	15.2 ± 6.7	14.5 ± 4.8	nd	0.010
32	α-himachalene	1.8 ± 1.8	3.9 ± 2.1	18.4 ± 6.1	9.6 ± 7.1	11.4 ± 7.5	4.4 ± 0.8	3.2 ± 3.3	nd	nd	0.030
33	α-humulene	nd	9.0 ± 6.5	1.9 ± 1.5	2.3 ± 2.3	1.0 ± 1.0	3.0 ± 1.0	32.0 ± 28.6	1.0 ± 0.2	nd	0.005
34	α-muurolene	nd	nd	5.6 ± 2.9	nd	nd	nd	nd	3.8 ± 1.0	nd	0.001
35	α-ylangene	nd	10.0 ± 3.3	nd	nd	13.6 ± 3.7	nd	nd	nd	nd	0.007
36	β-bergamotene	nd	nd	13.2 ± 6.1	nd	nd	nd	nd	nd	nd	0.001
37	β-bourbonene	nd	58.3 ± 43.3	108.2 ± 103.0	51.4 ± 52.7	116.8 ± 67.6	25.9 ± 23.9	31.2 ± 26.9	34.2 ± 6.6	1.0 ± 1.0	0.032
38	β-cedrene	nd	nd	30.9 ± 7.2	nd	30.0 ± 16.7	13.9 ± 4.6	nd	nd	nd	0.003
39	β-elemene	1.2 ± 1.0	17.2 ± 10.5	12.0 ± 11.8	18.9 ± 16.3	26.3 ± 4.7	3.4 ± 2.4	30.2 ± 25.3	3.7 ± 1.4	nd	0.014
40	β-gurjunene	nd	2.5 ± 1.7	nd	6.0 ± 1.0	nd	nd	nd	8.9 ± 3.0	nd	0.008
41	β-santalene	nd	5.0 ± 2.1	6.2 ± 2.2	13.0 ± 8.9	13.7 ± 4.6	nd	nd	7.9 ± 1.6	nd	0.011
42	γ-cadinene	nd	5 ± 1	4 ± 1	nd	nd	nd	nd	nd	nd	0.090
43	γ-muurolene	nd	nd	10.2 ± 2.3	12.9 ± 9.5	nd	4.9 ± 2.0	3.8 ± 3.7	2.2 ± 0.4	nd	0.014
44	δ-cadinene	nd	10.2 ± 0.7	nd	nd	nd	nd	nd	nd	nd	0.007
	Total sesquiterpenes	18.5 ± 6.3	424.2 ± 268.7	431.1 ± 229.0	341.4 ± 258.2	388.3 ± 155.7	158.1 ± 103.8	227.2 ± 186.7	141 ± 26.8	4.3 ± 1.6	0.000
	Total terpenoids	1195.9 ± 220.5	1112.4 ± 289.0	1819.8 ± 722.9	1498.8 ± 701.0	1360 ± 506.9	1859.4 ± 752.5	1645.3 ± 698.1	1464.7 ± 232.5	828.7 ± 73.1	0.713
	**Geranylgeranyl diphosphate (GGDP) pathway products**										
45	6-methyl-5-hepten-2-one	5.7 ± 3.1	4.8 ± 1.0	6.8 ± 5.4	4.7 ± 1.8	7.7 ± 6.0	6.9 ± 1.7	8.1 ± 3.2	10.2 ± 1.1	4.7 ± 0.4	0.823
	**Short-chained oxygenated volatiles (OVOC)**										
46	acetaldehyde	nd	nd	nd	107.9 ± 36.0	nd	nd	46.7 ± 15.6	nd	38.6 ± 2.8	0.000
	**Total VOCs**	1237.8 ± 233.1	1150.9 ± 295.2	1892.4 ± 765.3	1641.3 ± 737.7	1431.5 ± 537.8	1915.8 ± 764.8	1792.4 ± 731.7	1517.1 ± 213.4	888.4 ± 97.6	0.721

nd: non-detected (equated to zero in the Kruskal–Wallis test). *p*-value (considered significant at *p* < 0.05).

**Table 3:** Principal component analysis (PCA) based on the volatile organic compound emissions of nine potato cultivars with different late blight resistance: individual component loadings and percentage of total variation explained by PCA axes, followed by a heatmap scale of PCA loadings; the areas highlighted in green represent positive loadings and those in red represent negative loadings.

		PC1	PC2	PC3	PC4
	**Eigenvalues**	10.71	8.96	7.11	5.98
	**% variation**	23.27	19.47	15.47	13
	**commulative%**	23.27	42.74	58.21	71.21
			**Loading**		
	**Lipoxygenase pathways (LOX) volatiles**				
1	1-hexanol	0.423095	0.873932	0.183125	−0.02479
2	hexanal	0.325118	0.889722	0.246182	0.148981
	**Long-chained saturated fatty acid-derived (FAD) compounds**				
3	decanal	0.116949	0.964819	−0.06845	0.19238
4	heptanal	0.19379	0.895935	0.101792	0.36117
5	nonanal	0.207871	0.942118	0.065361	0.066343
6	octanal	0.283918	0.935551	0.062605	0.152954
7	**Isoprene**	0.140161	−0.27586	0.819493	0.014697
	**Monoterpenes**				
8	camphene	0.961397	−0.08033	0.037266	−0.08403
9	camphor	0.154466	0.39425	0.672138	−0.3839
10	eucalyptol	−0.18351	−0.19783	−0.08726	−0.02872
11	limonene	0.914702	0.266727	0.091243	−0.18888
12	*p*-cymene	0.946021	0.061377	0.04605	−0.0309
13	tricyclene	0.467093	−0.31973	0.102338	0.121303
14	α-fenchene	0.93641	0.02269	0.062651	−0.01463
15	α-pinene	0.937804	0.255374	0.076056	−0.10775
16	α-terpinene	−0.44437	−0.08688	−0.04666	−0.03485
17	α-thujene	0.780768	0.497105	0.178233	−0.03042
18	β-myrcene	0.239572	0.132158	0.195393	0.12115
19	β-phellandrene	0.785454	0.513017	−0.05613	−0.23451
20	β-pinene	0.866342	0.416421	0.21344	−0.09975
21	Δ^3^-carene	0.903693	0.359793	0.119169	−0.13791
	**Sesquiterpenes**				
22	(*E*)-β caryophyllene	−0.29777	−0.04812	0.012009	0.090055
23	(*E*)-β-farnesene	−0.15699	0.083463	−0.11748	0.89168
24	longifolene	0.721781	0.093535	0.431241	−0.06346
25	thujopsene	−0.22349	0.222433	−0.16337	0.866308
26	valencene	−0.19777	0.045587	0.797613	−0.00057
27	α-bergamotene	0.000158	0.73073	0.555791	0.18202
28	α-caryophyllene	0.806852	−0.18564	−0.26937	0.077299
29	α-copaene	0.229122	0.07233	0.916447	0.283393
30	α-cubebene	0.003861	0.130598	0.191692	0.94465
31	α-gurjunene	−0.06896	0.252143	0.115276	−0.16672
32	α-himachalene	0.044749	0.211	0.710516	0.537144
33	α-humulene	−0.02824	0.850289	−0.24035	−0.35799
34	α-muurolene	0.211352	−0.01158	0.854113	0.000106
35	α-ylangene	−0.4728	0.135766	−0.16961	0.720814
36	β-bergamotene	0.098933	0.11782	0.966903	0.025226
37	β-bourbonene	−0.11645	0.301694	0.515712	0.77391
38	β-cedrene	0.189365	0.192938	0.529119	0.695526
39	β-elemene	−0.25946	0.77921	−0.09361	0.393419
40	β-gurjunene	0.057422	−0.33093	−0.04696	0.033654
41	β-santalene	−0.20411	−0.06907	0.154352	0.785958
42	γ-cadinene	−0.26991	0.020038	0.678889	−0.00825
43	γ-muurolene	0.352984	0.01701	0.462227	0.049684
44	δ-cadinene	−0.44437	−0.08688	−0.04666	−0.03485
	**GGDP pathway products**				
45	6-methyl-5-hepten-2-one	0.440136	0.410775	0.021837	0.175131
	**Short-chained oxygenated volatiles (OVOC)**				
46	acetaldehyde	−0.16219	0.008224	−0.22616	−0.13996

## Data Availability

Not applicable.

## Supplementary Materials

The following supporting information can be downloaded at: https://www.mdpi.com/article/10.3390/plants12112100/s1. Figure S1: Biplot of the first two principal components of the different VOC classes showing the distribution of different potato cultivars. (a) long-chained saturated-fatty-acid-derived compounds, including classic green leaf volatile, (b) monoterpenes, (c) sesquiterpenes, (d) terpenoids. Refer to Table 2 for numbers assigned to each VOC. Table S1: Principal component analysis (PCA) based on the volatile organic compound emissions by nine potato cultivars with different late blight resistance, eigenvalues, and percentage of total variation explained by PCA axes, followed by heatmap scale of PCA loadings, highlighted green represents positive loadings and red represents negative loadings.
