# Supplementary material for: Variation in Leaf Volatile Emissions in Potato (Solanum tuberosum) Cultivars with Different Late Blight Resistance
Source: Plants (Basel). 2023 May 25;12(11):2100. doi: 10.3390/plants12112100 (PMC10255465; doi:10.3390/plants12112100)
Supplement: Supplementary file 1 [file plants-12-02100-s001.zip › Supplementary Figure S1.pdf]

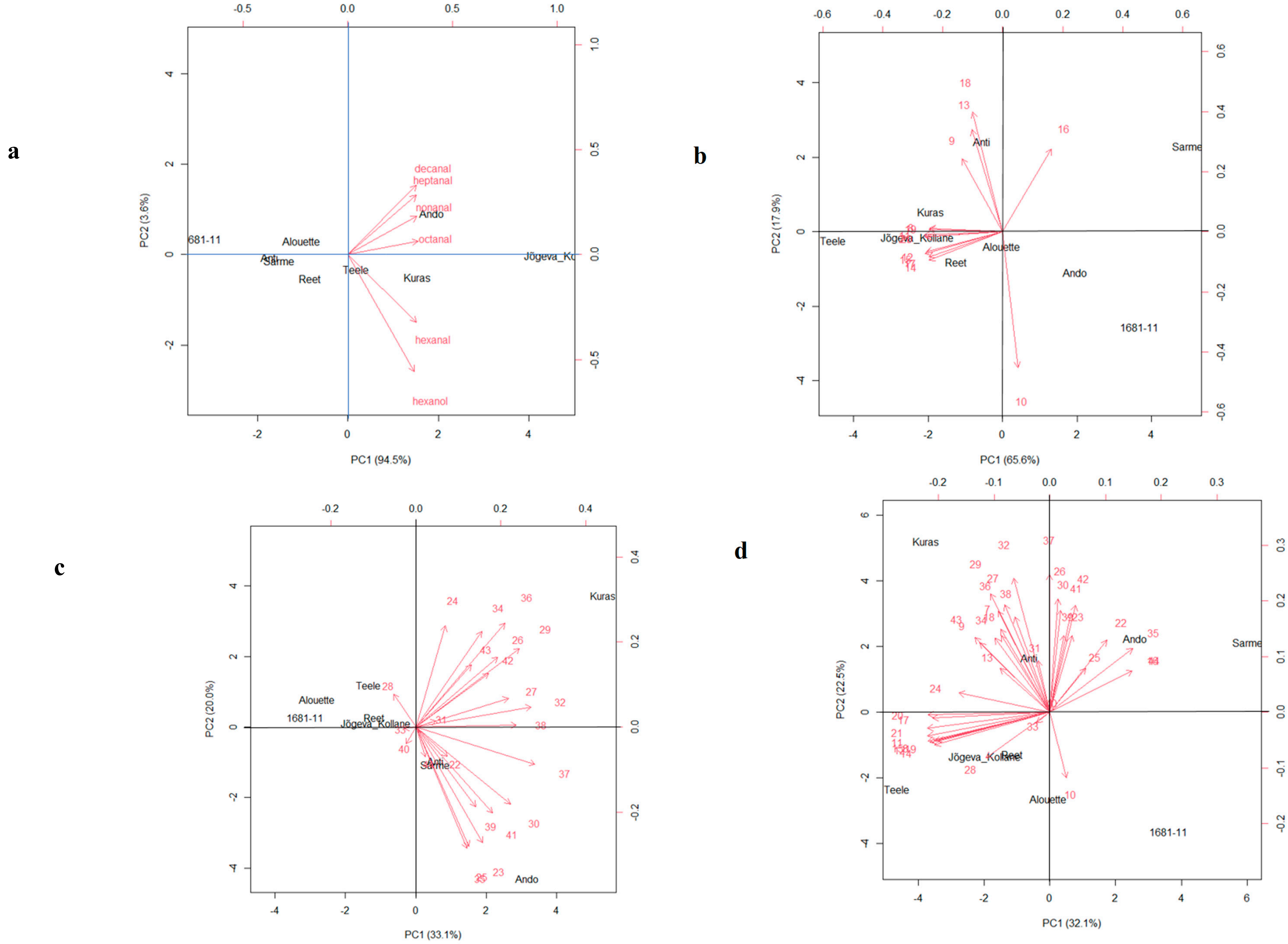

Figure S1. Biplot of the first two principal components of the different VOC classes showing the distribution of different potato cultivars. (a) long-chained saturated-fatty-acid-derived compounds, including classic green leaf volatile, (b) monoterpenes, (c) sesquiterpenes, (d) terpenoids. Refer to Table 2 for numbers assigned to each VOC.
